# Epidemiology, Diagnosis, and Management of Cystic Lesions of the Pancreas

**DOI:** 10.1155/2012/147465

**Published:** 2011-10-11

**Authors:** Koen de Jong, Marco J. Bruno, Paul Fockens

**Affiliations:** ^1^Department of Gastroenterology and Hepatology, Academic Medical Center, University of Amsterdam, P.O. Box 22700, 1100 DE Amsterdam, The Netherlands; ^2^Department of Gastroenterology and Hepatology, Erasmus Medical Center, 3000 CA, Rotterdam, The Netherlands

## Abstract

Although little is known on the true prevalence of pancreatic cysts, physicians are currently more frequently confronted with pancreatic cysts because of the increasing use of sophisticated cross-sectional abdominal imaging. Cystic lesions of the pancreas comprise of a heterogeneous group of diagnostic entities, some of which are benign such as inflammatory pseudocysts or serous cystadenomas and do not require resection when asymptomatic. Others like mucinous cysts or intraductal papillary mucinous neoplasms (IPMN) have a malignant potential and in these cases surgical resection is often indicated. For this reason an adequate distinction between the various cysts is crucial to optimize management strategy. Different diagnostic methods that could be of value in the differentiation include radiologic imaging techniques such as CT, MR, and endosonography. In addition, fluid aspiration for cytopathology, tumormarkers or molecular analysis is widely used. Different guidelines are available but so far no optimal diagnostic algorithm exists. We summarize the epidemiology, classification, clinical presentation, diagnostics, management, and future perspectives.

## 1. Introduction

As a result of the widespread use of cross-sectional imaging, clinicians are confronted with pancreatic cysts with increasing frequency [1]. The majority of these cysts are asymptomatic, and the decision whether or not to operate is not always straightforward. Although our knowledge of the pathophysiology and pathobiology of pancreatic cysts is increasing, relatively little is known about their natural history.

The apparent question is how to proceed after the detection of an asymptomatic pancreatic cyst choosing one of the following options: no further investigations, additional imaging ± fine needle aspiration (FNA), surveillance, or surgical/endoscopic treatment. Despite a spectacular improvement in diagnostic modalities in the past decades, differential diagnosis and hence management of pancreatic cysts remain controversial. Most centers have adopted a differential approach with follow up in case of absence of secondary features of malignancy and surgical resection in case of a high suspicion of malignancy. Multiple guidelines have appeared. In this paper we will attempt to provide a comprehensive overview of the epidemiology, diagnostic options, and management of pancreatic cysts.

## 2. Epidemiology

**Figure 1:**
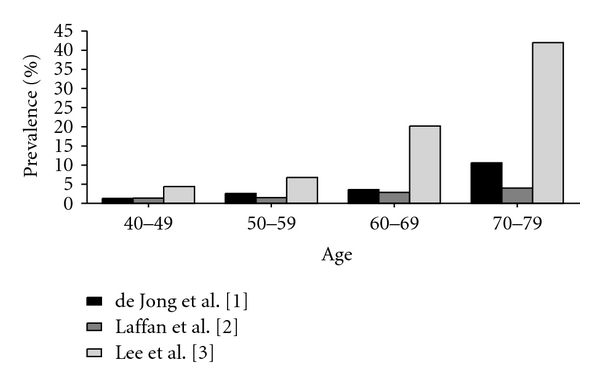
Prevalence of pancreatic cysts in relation to increasing age.

To date only a few studies have been performed investigating the true prevalence of pancreatic cysts. We have recently published a study in which 2803 magnetic resonance imaging (MRI) examinations were retrospectively reviewed in a group of mostly asymptomatic patients who decided to undergo a preventive screening abdominal MRI at their own initiative and costs without referral of a physician. Prevalence was 2.4% and increased with age [1]. A study by Laffan et al. reported a prevalence of 2.6% [2]. In retrospect, 2832 consecutive computed tomography (CT) scans were reviewed. Patients with known pancreatic disease or symptoms related to the pancreas were excluded. A prevalence of 13.5% was found in another recent retrospective study in 616 patients using MRI [3]. Patients were excluded from this study if they had a known or suspected history of pancreatic disease. In all these studies increasing age correlated with a higher prevalence of pancreatic cysts (Figure 1).

In an older Italian study reports of 24,039 MRI and CT scans were retrospectively reviewed with a computerized search. Pancreatic cysts were reported in 1.2% of which 58% (0.7% of total study population) did not have a history of pancreatitis [4]. The highest prevalence of pancreatic cysts using a radiologic imaging technique was found in a study by Zhang et al. [5]. Spin-echo MR images of 1444 patients were reviewed for pancreatic cysts by two radiologists, and pancreatic cysts were described in 19.6% of patients. Patients with known history of pancreatic disease were not excluded from this study.

In an autopsy study of 300 cases a stunning 24.3% were found to have pancreatic cysts [6]. It is of note that this study was performed in elderly patients (more than 80% were older than 65 years), and no information was provided of a possible history of pancreatic disease. The results of the described studies are summarized in Table 1. The broad range of prevalence values can be explained by the fact that studies differed in the selection of the study population, in-hospital or out-patient based and whether patients with potential pancreatic disease were excluded from analysis. Importantly, studies also differed in which imaging modality was employed with each technique having its distinct sensitivity and specificity for detecting cysts.

## 3. Classification of Pancreatic Cystic Lesions

### 3.1. Nonneoplastic Pancreatic Cysts

The most common nonneoplastic pancreatic cysts are serous cystadenomas and pancreatic pseudocysts, and these types are described in more detail in this paper. Rare nonneoplastic pancreatic cysts include true cysts, retention cysts, and lymphoepithelial cysts.

#### 3.1.1. Serous Cystadenoma

Patients with serous cystadenomas (SCNs) are predominantly elderly women with a median age of approximately 60 years, and the cysts can arise in any region of the pancreas.

Classical features of a serous cystadenoma include microcystic morphology, a central area of calcification, and a watery, nonviscous fluid content. However a macrocystic variant of serous cystadenomas exists and can easily be confused with a pseudocyst or a mucinous cystadenoma [7–9]. Serous cystadenomas are lined by a glycogen-rich cuboidal epithelium which can be shown with cytopathological analysis [10]. Although a small number of cases of malignant serous cystadenocarcinomas have been described, it is generally believed that serous cystadenomas have virtually no malignant potential [11]. Serous cystadenomas can be treated conservatively if the patient is asymptomatic. Surgery is treatment of choice when a patient has symptoms or the distinction between a serous cystadenoma and a mucinous cystic neoplasm is not possible. 

#### 3.1.2. Pseudocysts

Pancreatic pseudocysts are fluid collections arising from leakage of the pancreatic duct lacking an epithelial lining. They usually occur following the course of an acute pancreatitis, chronic pancreatitis or secondary to an abdominal trauma [12]. The incidence of pseudocysts in the phase of an acute pancreatitis is 5.1% to 16% [13–15] whereas the incidence in chronic pancreatitis is higher with percentages varying from 20% to 40% [16–18]. 

Radiologic imaging of pseudocysts frequently shows a single cystic lesion, without septations or solid components. Aspirated fluid often has a low viscosity, high amylase, and cytology which is consistent with an inflammatory origin. The cysts are often filled with protease-free serous fluid if no connection to the pancreatic duct exists. Whereas size of >6 cm and duration of more than 6 weeks used to be main indicators for intervention, currently symptomatology is the main indicator for intervention.

### 3.2. Neoplastic Pancreatic Cysts

The majority of neoplastic cysts are represented by mucinous cystic neoplasms (MCNs) (10–49%) and intraductal papillary mucinous neoplasm (IPMN) (21–33%) [19, 20]. Solid pseudopapillary neoplasms are less common. Other rare neoplastic cystic lesions include cystic neuroendocrine tumors and acinar cell cystadenocarcinomas but these will not be discussed in this paper.

#### 3.2.1. Mucinous Cystic Neoplasm

Patients with MCNs are almost exclusively middle-aged women [21, 22], and most of the MCNs appear in the body or tail of the pancreas although they occasionally may occur in the head. The average size of the cysts is larger than 5 cm at time of presentation [22–24]. MCNs are generally macrocystic, thick-walled cysts that typically lack communication with the ductal system [25, 26]. A microcystic MCN is rarely seen [27, 28]. They are either unilocular or multilocular with a small number of compartments [29]. Unique is the fact that MCNs contain a mucinous, dense ovarian stroma surrounding the epithelial cells, which is never seen in other cystic lesions. Therefore, ovariantype stroma is considered a requisite to distinguish MCNs from the other cystic neoplasms.

#### 3.2.2. Intraductal Papillary Mucinous Neoplasms

IPMNs are slightly more often seen in male patients and they are usually older at presentation than patients with MCNs or serous cystadenomas. Most of the IPMNs arise in the head and uncinate process of the pancreas, and they are typically connected to the ductal system of the pancreas. IPMNs comprise lesions of the main pancreatic duct, side branches or a combination of these two. They have mixed features of microcystic and macrocystic lesions, and the main pancreatic duct is often dilated. IPMNs contain mucinous fluid which is sometimes extruding from the ampulla of Vater. An important difference in prevalence of malignancy exists for main-duct and side-branch IPMNs. The prevalence of malignancy for lesions of the main-duct IPMN is 57–92% whereas it is 6–46% for lesions of side-branch IPMN [30].

#### 3.2.3. Solid Pseudopapillary Neoplasms

Solid pseudopapillary neoplasms (SPNs) are rare lesions which make up 1-2% of all pancreatic cystic neoplasms [31, 32]. They are almost exclusively found in young women with a median age of 30 years [33–35]. On the basis of the largest review [36], tumors ranged in size from 0.5 to 34.5 cm with a mean diameter of 6.08 cm.

They are equally distributed throughout the pancreas [36]. Solid pseudopapillary neoplasms often start as solid tumors and undergo degeneration giving it a cystic appearance on radiologic imaging [34]. On CT and MRI, the tumor is often well circumscribed, encapsulated, and heterogeneous with hemorrhagic and cystic degeneration [32]. Solid pseudopapillary neoplasms are tumors with relatively low malignant potential, with a reported incidence of malignant transformation of 15% [34]. Surgical resection of distant metastases is justified due to the excellent long-term prognosis in the presence of metastatic disease [37]. Characteristics of different pancreatic cysts are summarized in Table 2.

## 4. Clinical Presentation

Many patients with cystic lesions of the pancreas present without abdominal complaints [38]. Lesions are often detected when a radiologic examination is performed for another reason or when an individual decides to undergo preventive screening investigations. When the pancreatic cyst is symptomatic, patients may present with epigastric pain, postprandial fullness, palpable mass, gastric outlet obstruction, nausea, vomiting, diarrhoea, steatorrhea, and/or weight loss. Patients with IPMNs sometimes present with recurrent episodes of pancreatitis. Side-branch IPMNs are more often asymptomatic than main-duct IPMNs. MCNs and pseudopapillary neoplasms are frequently large at time of diagnosis and symptoms are more common in these patients. When an advanced cystic neoplasm exists, patients often present with complaints similar to pancreatic adenocarcinoma such as pain, weight loss, and jaundice [39].

## 5. Diagnostics

**Figure 2:**
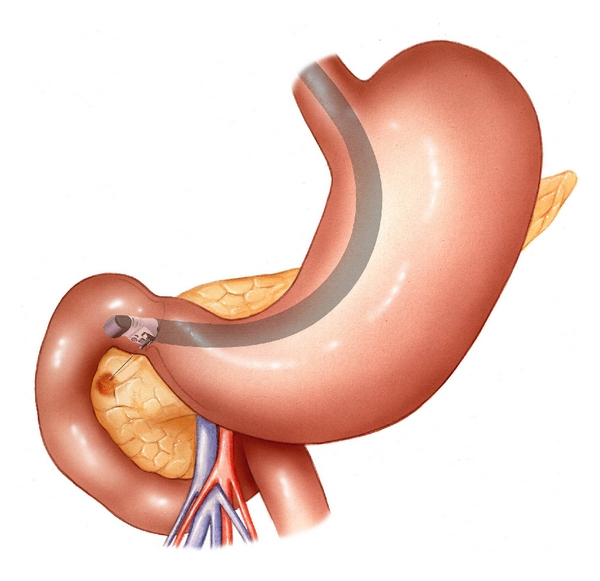
Cystic lesion in the pancreatic head is punctured using a linear array echo-endoscope.

**Figure 3:**
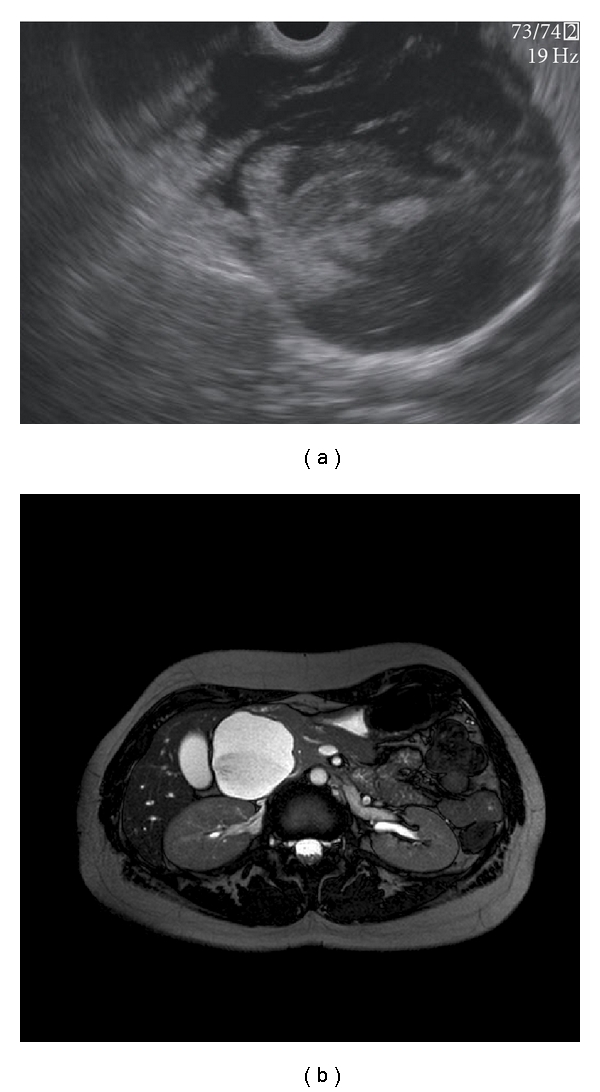
(a) EUS image of a malignant IPMN in the head of the pancreas. (b) MRI image of a malignant IPMN in the head of the pancreas.

**Figure 4:**
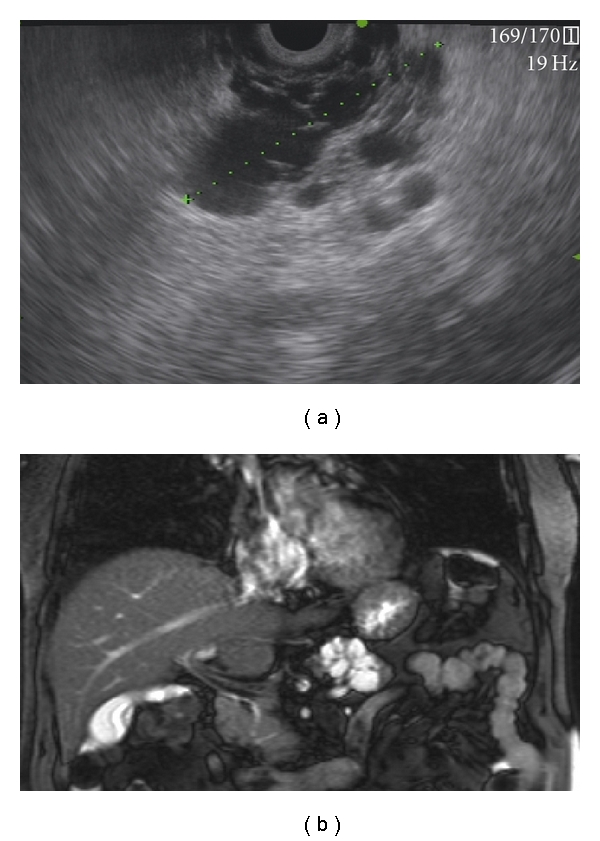
(a) EUS image of a serous cystadenoma in the head of the pancreas. (b) MRI image of a serous cystadenoma in the head of the pancreas.

Diagnostic methods that can be valuable in the differentiation of pancreatic cysts include radiologic imaging techniques such as abdominal ultrasound (US), computed tomography (CT), and magnetic resonance imaging (MRI). Endoscopic ultrasonography (EUS) and EUS-guided fine needle aspiration (EUS-FNA) for cytopathologic examination, tumormarker determination, and molecular analysis are also widely used (Figures 2, 3, and 4).

Transabdominal ultrasonography is a safe imaging technique without radiation exposure which is helpful in the differentiation of solid and cystic lesions. It is currently widely used in the evaluation of abdominal complaints. As a result, cystic lesions are often initially detected with this modality. It is however not the imaging of first choice since it is difficult to visualize the complete pancreas due to overlying bowel or fat, and it is rather operator dependent. CT is often used in the diagnostic workup. It is a widely used imaging technique to visualize and differentiate pancreatic cysts based on morphologic features as size, microcystic/macrocystic aspect, presence of septations, nodules, and calcifications [40, 41]. MRI has the additional advantage to show a possible connection with the pancreatic duct which on T2-weighted image sequences is better visualized than with CT [42]. Another advantage of MRI, especially for follow up of the cysts, is the lack of radiation exposure. 

EUS has emerged as a useful diagnostic technique in the evaluation of pancreatic cystic lesions, providing fine detail on the characteristics of the cyst because of the very high spatial resolution. It has therefore been suggested as an ideal imaging technique for pancreatic cysts [27, 43–45]. EUS can image characteristics of the cysts as well as the parenchymal changes and has a role in determining the resectability if malignancy is present [46]. Despite the fact that EUS is presently widely used for the differential diagnosis, a number of points of discussion still exist. Since EUS is invasive, technically difficult, and expensive, it is not available in all hospitals. Furthermore there is a substantial interobserver agreement between endosonographers. In a multicenter study 8 experienced endosonographers reviewed videotapes of 31 EUS procedures of pancreatic cysts. In this study there was only poor to moderate agreement for the diagnosis of neoplastic versus nonneoplastic, specific type, and EUS features [47]. An advantage of EUS is the possibility to perform FNA for analysis of the cyst fluid. EUS-FNA is considered a safe technique to obtain pancreatic cyst fluid with rare, mostly mild complications, but infection, pancreatitis, and intracystic haemorrhage have been reported [48, 49]. Infection of cysts after FNA is rare and, although common practice in most centers, data are lacking to support the use of prophylactic antibiotics. Furthermore, to minimize the risks of subsequent infection one should keep the number of punctures to a minimum and attempt to aspirate all fluid from the cyst whenever possible. Intracystic hemorrhage is a complication that occurred in 6% of all cases reported by Varadarajulu et al. but most of the complications were mild and did not need further medical intervention [50]. 

Cytological evaluation of pancreatic cyst fluid is widely used, and several studies report a sensitivity of approximately 50% for the differentiation of mucinous and nonmucinous pancreatic neoplasms [51–53]. However, other studies show less positive results since cytopathology is often nondiagnostic due to the low cellularity of the obtained cyst fluid [54, 55]. Biochemical analysis of cyst fluid and tumor markers have been evaluated for several years with the underlying thought that markers secreted into the cyst fluid identify the epithelial lining. Amylase is usually elevated in pseudocysts and IPMNs and low in MCNs and serous cystadenomas. Of the tumor markers, CEA is considered the best discriminant marker to differentiate between a mucinous and a nonmucinous cyst [54, 56]. A low CEA level (<5 ng/mL) has been shown to have a sensitivity between 50% and 100% and a specificity of 77–95% to differentiate between mucinous and nonmucinous cysts [51]. Pseudocysts and serous cystadenomas generally have a low CEA value. Currently, the most widely used cutoff for an elevated CEA is 192 ng/mL, which was established in a study by Brugge et al. as diagnostically sensitive in 75% and specific in 84% to discriminate between mucinous and nonmucinous cysts [54]. Altogether, the current yield of FNA is small, which can be caused by the microcystic aspect of a cyst, the high viscosity of the fluid or the minimum amount of fluid that is needed for certain examinations of the fluid. The standard use of a 19 G needle could be helpful to aspirate both larger cysts and cysts which contain fluid with a high viscosity.

## 6. Management (Guidelines)

The most recent guideline for the management of pancreatic cyst was published in 2007 by Khalid and Brugge [57]. In this guideline the authors advice to thoroughly evaluate each incidental pancreatic cyst since many cysts are premalignant (MCN and IPMN). The initial imaging test proposed is a contrast-enhanced triphasic multidetector CT scan, which may be followed by EUS-FNA in particular cases when FNA is needed for CEA level or to puncture a solid component. Resection is recommended in all MCNs and main-duct IPMNs. Firm recommendations for the management of branch-duct IPMNs are not provided. Serous cystadenomas should only be resected if symptomatic or if the diagnosis remains in doubt. All pseudopapillary neoplasms should be considered for resection. No general guidelines are provided for the interval of follow up when surgery is not undertaken. The authors state that this decision depends on the kind of lesion and the reason why surgery was not performed.

The American Society for Gastrointestinal Endoscopy issued a guideline on the use of EUS in the management of pancreatic cysts [58]. Cystic lesions of the pancreas require diagnostic evaluation regardless of size, and EUS alone is considered not accurate enough to definitively diagnose the type of cystic lesion or to determine its malignant potential. Furthermore, FNA is advised with a low sensitivity of cytologic analysis but a high specificity for MCN and malignancies. Biochemical analysis may provide clinically useful information but cannot provide a definitive diagnosis or determine whether the lesion is malignant. In this guideline it is stated that there are currently no accepted endoscopic therapies for cystic neoplasms of the pancreas, and there is a role for endoscopic drainage of inflammatory pancreatic fluid collections. 

In 2005 international consensus guidelines for the management of IPMNs and MCNs were published in which a list of clinically relevant questions and answers is provided [30]. The recommendation is to resect all main-duct and mixed variant IPMNs regardless of size as long as the patient is a good surgical candidate. Asymptomatic side-branch IPMNs can be followed with CT or MRI as long as there are no mural nodes, dilatation of the main duct or growth in size. The authors do not explicitly state that all branch-duct IPMNs >3 cm should be resected. More data based on branch-duct IPMNs >3 cm without main-duct dilatation or mural nodules are needed to determine if all branch-duct IPMNs >3 cm should be resected immediately. The authors state that MCNs should always be resected unless there are contraindications for surgery.

## 7. Future Developments

New methods to improve the yield of FNA are urgently required. Existing tumor markers have only limited value, and more sensitive biomarkers need to be identified. New techniques including proteomics and molecular analysis may be helpful for the differential diagnosis of pancreatic cysts [59]. 

Also the development of new techniques to minimize the fluid needed for examinations may well be useful. Furthermore, the development of new techniques to increase the cellularity of the obtained fluid could be helpful. Three reports have been recently published, studying a new type of brush (EchoBrush, Cook Medical) to improve the yield of cytologic examination [60–62]. These studies suggest that this relatively new technique improves the yield, but larger randomized trials are necessary to confirm these results and to define the safety profile of this more aggressive approach. 

Currently, no accepted endoscopic treatment option for neoplastic cystic lesions is available but a few experimental studies have been performed to determine the safety and effectiveness of EUS-guided ethanol lavage with paclitaxel to treat pancreatic cysts [63–65]. The first studies report that this technique is a safe and feasible but larger studies with longer follow up are necessary.

## 8. Conclusion

Patients presenting with pancreatic cysts have to be thoroughly evaluated. Cross-sectional imaging should be used for the morphological characterization, and EUS-FNA for fluid and tissue sampling could be used in particular cases to discriminate between mucinous and nonmucinous cysts. Management should be based upon on carefully weighting the malignant potential of a pancreatic cystic lesions and the risk of surgery. Larger prospective studies with longer follow up are needed to increase the knowledge of the natural history of pancreatic cysts.

## Figures and Tables

**Table 1:** Characteristics of studies on pancreatic cyst prevalence.

Study	Number of patients	Prevalence (%)	Technique	Patients with known pancreatic disease excluded
de Jong et al. [1], 2010	2803	2.4	MRI	Yes
Laffan et al. [2], 2008	2832	2.6	CT	Yes
Lee et al. [3], 2010	616	13.5	MRI	Yes
Spinelli et al. [4], 2004	24039	1.2	MRI and CT	No
Zhang et al. [5], 2002	1444	19.6	MRI	No
Kimura et al. [6], 1995	300	24.3	autopsy	No

**Table 2:** Characteristics of different pancreatic cysts.

	MCN	IPMN	SPN	SCN	Pseudocyst
Sex distribution	F > M	M = F	F > M	F > M	F = M
Age	40–60	60–70	20–30	60–70	All ages
Average size of cyst	>3 cm	<3 cm	>3 cm	>3 cm	>3 cm
Morphologic characteristics	Septations thickened wall macrocystic	Dilatation of PD micro/macrocystic	Mixed solid and fluid with hemorrhage	Microcystic	Unilocular thick wall
Fluid	Viscous, clear	Viscous, clear	Thin, bloody	Thin, clear	Thin, dark
Malignant potential	Yes	Yes	Yes	No	No

MCN: mucinous cystic neoplasm, IPMN: intraductal papillary mucinous neoplasm, SPN: solid pseudopapillary neoplasm, SCN: serous cystic neoplasm, PD: pancreatic duct.
